# Patient stakeholder engagement in research: A narrative review to describe foundational principles and best practice activities

**DOI:** 10.1111/hex.12873

**Published:** 2019-02-13

**Authors:** James D. Harrison, Andrew D. Auerbach, Wendy Anderson, Maureen Fagan, Martha Carnie, Catherine Hanson, Jim Banta, Gina Symczak, Edmondo Robinson, Jeffrey Schnipper, Celene Wong, Rachel Weiss

**Affiliations:** ^1^ Division of Hospital Medicine University of California San Francisco San Francisco California; ^2^ University of Miami Health System Miami Florida; ^3^ Center for Patients and Families Brigham and Women’s Hospital Boston Massachusetts; ^4^ Intensive Care Unit Patient and Family Advisory Council University of California San Francisco San Francisco California; ^5^ Department of Medicine Christiana Care Health System Wilmington Delaware; ^6^ Department of Medicine Brigham and Women’s Hospital Boston Massachusetts

**Keywords:** patient engagement, patient involvement, patient participation, research, review, systematic review

## Abstract

**Background:**

Health research is evolving to include patient stakeholders (patients, families and caregivers) as active members of research teams. Frameworks describing the conceptual foundations underlying this engagement and strategies detailing best practice activities to facilitate engagement have been published to guide these efforts.

**Objective:**

The aims of this narrative review are to identify, quantify and summarize (a) the conceptual foundational principles of patient stakeholder engagement in research and (b) best practice activities to support these efforts.

**Search Strategy, Inclusion Criteria, Data Extraction and Synthesis:**

We accessed a publicly available repository of systematically identified literature related to patient engagement in research. Two reviewers independently screened articles to identify relevant articles and abstracted data.

**Main Results:**

We identified 990 potentially relevant articles of which 935 (94.4%) were excluded and 55 (5.6%) relevant. The most commonly reported foundational principles were “respect” (n = 25, 45%) and “equitable power between all team members” (n = 21, 38%). Creating “trust between patient stakeholders and researchers” was described in 17 (31%) articles. Twenty‐seven (49%) articles emphasized the importance of providing training and education for both patient stakeholder and researchers. Providing financial compensation for patient stakeholders’ time and expertise was noted in 19 (35%) articles. Twenty articles (36%) emphasized regular, bidirectional dialogue between patient partners and researchers as important for successful engagement.

**Discussion and Conclusions:**

Engaging patient stakeholders in research as partners presents an opportunity to design, implement and disseminate patient‐centred research. This review creates an overarching foundational framework for authentic and sustainable partnerships between patient stakeholders and researchers.

## 1 INTRODUCTION

As the people who directly experience illness and medical care, patients, families and caregivers are uniquely positioned to contribute to research efforts seeking to understand and improve illness diagnosis and management, treatments and health‐care delivery.^1^ Six levels of patient stakeholder (patients, families and caregivers) engagement in health research have been proposed ranging from patients as research subjects, to more collaborative relationships whereby patients are equal partners on a research team or even leading research teams.^2^ Health and medical research is rapidly evolving to include patient stakeholders as active members of research teams as advisors, collaborators and co‐investigators.

The value of including patient stakeholders in research includes improving the patient‐centredness of chosen research study design and outcomes, ensuring meaningful and culturally appropriate study materials as well as potential increases in recruitment and retention of study participants.^3^, ^4^, ^5^, ^6^ While improvements to the relevance and quality of research are important, more philosophical benefits include injecting democracy and accountability into the research process—especially for publicly funded research—and the empowerment of patient stakeholders.^7^, ^8^, ^9^, ^10^ These practical and moral arguments have led some funding agencies and journal editors to either mandate, or strongly endorse, patient stakeholder engagement in research including before grants and publications are considered for review.^4^, ^11^, ^12^, ^13^, ^14^

It is now established that engaging patient stakeholders in research is possible at all stages of the investigative process.^15^, ^16^ Areas where patient stakeholders can meaningfully contribute include agenda setting, research question prioritization, assistance during study implementation, review and interpretation of results, and dissemination.^15^, ^17^, ^18^ However, researchers attempting to engage and partner with patient stakeholders are faced with many challenges.^16^, ^19^, ^20^ This includes difficulty precisely defining roles and expectations of team members, and problems providing appropriate patient education materials about the research design and processes. Sub‐optimal, or nominal, engagement can result in tokenism where patient stakeholders left with the sense that their engagement is for the purpose of “checking a box” rather than for true partnership and collaboration*.* 19, 21 Many of these challenges are due to, or exacerbated by, researchers lacking expertise and skills in patient stakeholder engagement principles and activities.^17^

To address this, a number of frameworks describing the conceptual foundations of patient stakeholder engagement in research and strategies detailing best practice activities have been published to help guide researchers.^22^, ^23^, ^24^ This proliferation, although welcome, has led to a diffusion of information and has increased the choices for researchers as they seek to effectively operationalize patient stakeholder engagement. To date, there have been limited efforts to summarize or consolidate the key messages and information from these conceptual frameworks and best practice recommendations. Therefore, the aims of this narrative review are to identify, quantify and summarize (a) the conceptual foundational principles of patient stakeholder engagement in research and (b) best practice activities to support this type of engagement.

## 2 METHODS

We conducted a systematic review of the literature supplemented with review of the grey literature to create a narrative review addressing the aims of this study. We used the Preferred Reporting Items for Systematic Reviews and Meta Analyses (PRISMA) to guide the conduct and reporting of this review.^25^

### 2.1 Search strategy

We first accessed a publicly available repository of systematically identified articles created by the Patient Centered Outcomes Research Institute (PCORI) and Academy Health.^26^ The purpose of this repository is to systematically identify and collate English language articles from PubMED/MEDLINE that are related to patient stakeholder engagement in research. The repository identifies published articles from four specific areas namely: (a) the impact or effects of patient stakeholder in research; (b) research studies describing/exemplifying patient stakeholder in research; (c) evaluation strategies assessing patient stakeholder engagement; and (d) conceptual frameworks for patient stakeholder engagement in research. The search strategy for this repository was developed in partnership with a medical librarian given the poorly standardized nomenclature for patient stakeholder engagement in research in bibliographic databases. The specific terms and inclusion and exclusion criteria used for this repository are shown in Appendix S1A, B. The date range for the search was 01/01/1995 up until 07/27/17 (which is the date the review was conducted). Our second search strategy involved accessing the grey literature (eg Google Scholar) using terms such as “patient engagement” or “patient participation” and “biomedical research” or “clinical research” and also included websites of known organizations supporting patient stakeholder engagement in research (eg PCORI, the Alberta Strategy for Patient Oriented Research SUPPORT Unit [AbSPORU], the United Kingdom's National Institute for Health Research [NIHR], the Canadian Institutes of Health Research (CIHR) and the Institute for Patient & Family Centered Care [IPFCC]). The reference lists of articles and reports were also examined to identify any additional publications.

### 2.2 Study identification, data extraction and analysis

We selected articles for review if they detailed either (a) a framework describing the conceptual foundations of patient stakeholder engagement in research or (b) guidelines describing best practices for supporting engaging patient stakeholders in research. Articles were excluded if they (a) discussed projects that utilized patient stakeholders as partners in research but did not detail how this was accomplished, (b) discussed general consumer involvement in research or public health but did not specifically provide any guidance or any mechanisms or activities to support involvement, (c) only described patient stakeholder involvement in research prioritization or selection of research topics or outcomes, and (d) used community‐based participatory research methods but did not explicitly describe the conceptual principles or best practices for engaging stakeholders in research in this context.

We screened all titles and abstracts of all articles contained within the literature repository and identified in the grey literature. The full texts of potentially relevant articles or reports were then obtained. Two reviewers then independently extracted data on a standard data collection form (Appendix S2) to organize the information about authors, title, year of publication, country of origin, whether the article describes a framework or guidelines of best practices and a brief summary of framework or guidelines. The contents of articles were also reviewed to explicitly note which domains the authors considered foundational to patient stakeholder engagement in research. Similarly, activities that support the operationalization of engagement were also extracted. We then quantified the number of times each foundational principle and best practice was reported. Finally, we developed an overarching framework summarizing our key findings. The study team conceptualized three distinct but inter‐related elements of the patient stakeholder engagement in research process. This was achieved by thematically grouping the foundational principles and best practices identified.^27^

## 3 RESULTS

**Figure 1:**
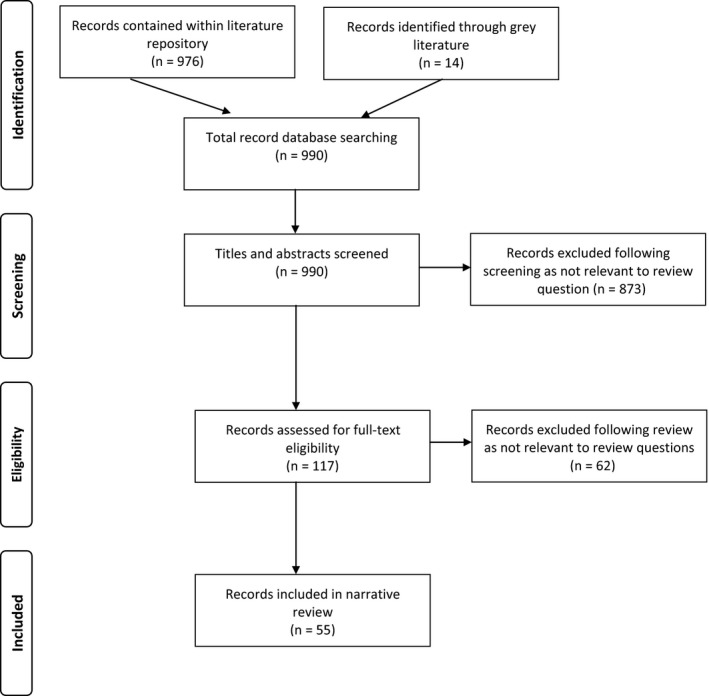
Review flow diagram

The article repository contained 976 potentially relevant articles and our search of the grey literature identified 14 additional articles (N = 990), of which 935 (94.4%) were excluded (see flow diagram Figure 1). The remaining 55 (5.6%) articles described the conceptual foundations of patient stakeholder engagement in research and best practice recommendations or activities to support these efforts.^5^, ^9^, ^13^, ^14^, ^17^, ^22^, ^23^, ^24^, ^28^, ^29^, ^30^, ^31^, ^32^, ^33^, ^34^, ^35^, ^36^, ^37^, ^38^, ^39^, ^40^, ^41^, ^42^, ^43^, ^44^, ^45^, ^46^, ^47^, ^48^, ^49^, ^50^, ^51^, ^52^, ^53^, ^54^, ^55^, ^56^, ^57^, ^58^, ^59^, ^60^, ^61^, ^62^, ^63^, ^64^, ^65^, ^66^, ^67^, ^68^, ^69^, ^70^, ^71^, ^72^, ^73^, ^74^ A full description of the 55 articles can be found in Appendix S3. Despite the distinction between frameworks and guidelines of best practices, we found considerable overlap in the content of article types: some solely describe a framework, others solely describe best practice activities, and the remainder describe a combination of both. North American authors followed by European authors published the majority of articles. Five articles describe the same framework as the basis for their discussion: for example, three utilize the PCORI Engagement Rubric, apply it in practice or suggest additional best practices based on lessons learned from using the model^5^, ^17^, ^22^ and two articles describe the FIRST model—a framework developed within rheumatology.^24^, ^38^ Also to note, the language used by authors to describe patient stakeholders varied considerably across articles and included terms such as patient stakeholders, patients, families, lay members, consumers and community members.

The foundational principles of patient stakeholder engagement in research and associated articles are delineated in Table 1. The most commonly reported principles were respect (n = 25, 45%) and equitable power between all team members (n = 21, 38%) including democratic and open forums in which all parties could express their views equally and without judgement. Creating trust between patient stakeholders and researchers was described in 17 (31%) articles as a core principle. Ensuring the diversity of patient stakeholders and inclusiveness (n = 12, 22%), promoting shared and collaborative decision making (n = 10, 18%) and open and transparent processes (n = 12, 22%) were also rated as important foundations to engagement efforts. Less frequently reported foundational principles related to maintaining the integrity of the engagement process and consciously maintaining confidentiality (n = 2, 4%).

**Table 1:** Summary of foundational principles of patient stakeholder engagement in research identified in 55 articles

Foundation principles	Article references	n (%)
Respect of stakeholders	9, 14, 17, 24, 28, 29, 32, 34, 35, 40, 43, 47, 48, 51, 53, 54, 56, 57, 58, 60, 62, 63, 69, 72, 74	25 (45)
Equitable power between stakeholders and researchers	9, 14, 17, 23, 24, 29, 32, 35, 38, 39, 40, 46, 50, 51, 53, 59, 63, 66, 68, 70, 71	21 (38)
Trust between stakeholders and researchers	5, 9, 22, 32, 35, 38, 40, 42, 45, 53, 55, 56, 60, 62, 63, 70, 72	17 (31)
Transparency/openness between stakeholders and researchers	5, 9, 22, 28, 29, 34, 35, 47, 48, 56, 57, 58	12 (22)
Ensuring diversity of stakeholders and inclusiveness	5, 14, 29, 46, 47, 48, 50, 56, 62, 64, 70, 72	12 (22)
Shared and collaborative decision making	5, 14, 23, 28, 32, 43, 45, 49, 60, 62	10 (18)
Support and flexibility of engagement process of activities	14, 47, 48, 51, 53, 55, 62, 68, 69	9 (16)
Honesty of research team	5, 9, 17, 22, 34, 58, 69	7 (13)
Support from institutional/organizational leadership	28, 45, 51, 56, 62, 69, 70	7 (13)
Promote ownership/empowerment	30, 34, 35, 43, 57, 58	6 (11)
Avoid tokenism	51, 62, 66, 69	4 (7)
Integrity of research team	53, 56, 61	3 (5)
Remaining conscious of confidentiality	66, 69	2 (4)
Responsiveness to act on patient stakeholder involvement/input	47, 48	2 (4)
Accountability between stakeholders and researchers to the wider community	47, 48	2 (4)

Articles also provided guidance and best practice recommendations of activities that support the realization of the foundational principles of patient stakeholder engagement in research (Table 2). Twenty‐seven (49%) articles emphasized the importance of providing access to training and education for both patient stakeholder partners—for example, in research methods—as well as researchers—particularly in how best to engage with patients and families. Providing financial compensation and reimbursement for patient stakeholders’ time, expertise and expenses was noted in 19 (35%) articles. In preparing for engagement, many articles noted that researchers should take the time to understand each patient stakeholder's skill set and experiences as this would optimize the contributions that they can make to a research project (n = 19, 35%). Twenty articles (36%) emphasized regular, bidirectional dialogue between patient partners and researchers as important for successful engagement. Less frequent but notable recommendations were to appoint a neutral facilitator during research meetings to reduce power differentials and facilitate open discussions between researchers and patient stakeholders (n = 5, 9%). Involving patient stakeholders early in research projects, creating a system for acknowledging and validating their contributions and developing processes for ongoing monitoring, evaluation and feedback also were described as worthwhile activities to promote engagement. In terms of structures to promote engagement in research, seven articles (13%) suggested the establishment of Patient and Family Advisory Councils (PFACs) as helpful, while two (4%) outlined the use of subcommittees within PFACs to focus on research activities.

**Table 2:** Summary of best practice activities to support patient stakeholder engagement in research identified in 55 articles

Best practice activity	Article references	n (%)
Training and education of researchers and patients	5, 13, 22, 23, 24, 28, 32, 35, 38, 39, 42, 43, 45, 46, 49, 51, 52, 54, 56, 59, 62, 63, 64, 65, 69, 71, 74	27 (49)
Regular dialogue/Bidirectional communication	5, 13, 24, 29, 39, 42, 50, 51, 54, 55, 56, 58, 62, 65, 66, 67, 69, 71, 72, 74	20 (36)
Compensation and reimbursement of out‐of‐pocket expenses	5, 24, 30, 31, 34, 38, 39, 43, 45, 49, 52, 57, 58, 60, 63, 64, 69, 70, 74	19 (35)
Select patient partners based on their skills and interests	23, 24, 31, 33, 34, 35, 38, 43, 45, 55, 58, 60, 62, 63, 67, 69, 71, 73, 74	19 (35)
Clarify roles of stakeholders	13, 17, 23, 24, 31, 34, 37, 38, 39, 44, 46, 51, 55, 56, 61, 62, 63, 69, 74	19 (35)
Ongoing monitoring and evaluation of engagement process	17, 24, 31, 32, 35, 38, 42, 50, 52, 58, 59, 62, 63, 65, 67, 70, 71, 73	18 (33)
Involve stakeholders early in research study	13, 23, 24, 34, 36, 37, 38, 43, 45, 50, 51, 57, 65, 66, 67, 68, 71, 74	18 (33)
Set and manage expectations/realistic goals	13, 17, 24, 31, 34, 36, 37, 38, 39, 49, 65, 67, 68, 71	14 (25)
Regular acknowledgement of stakeholder contributions	23, 24, 28, 35, 38, 41, 50, 57, 59, 61, 64, 66, 70, 74	14 (25)
Regular face‐to‐face/in‐person contact	24, 30, 33, 38, 43, 49, 58, 60, 74	9 (16)
Appoint a coordinator to manage engagement	24, 31, 33, 38, 45, 49, 52, 69, 74	9 (16)
Define scope of engagement for each project	13, 29, 31, 43, 49, 50, 61, 62, 69	9 (16)
Use lay language and avoid jargon	23, 32, 36, 37, 39, 52, 58, 62	8 (15)
Secure and budget for engagement activities	31, 32, 37, 56, 60, 63, 69, 74	8 (15)
Patient & Family Advisory Council (PFAC) model	17, 34, 38, 50, 54, 55, 74	7 (13)
Neutral facilitator/moderator	23, 31, 46, 55, 69	5 (9)
Accessible/regular meetings	24, 38, 46	3 (5)
Use experienced partners as support	34, 38, 39	3 (5)
Allow informal socializing/networking	58, 68, 69	3 (5)
Work in small groups	58, 60	2 (4)
Allow for subcommittees to work on	45, 63	2 (4)
Allow time to build relationships	51, 62	2 (4)
Maintain continuity of membership	68	1 (2)
Involve patient organizations	44	1 (2)
Hire staff from community of study	69	1 (2)

**Figure 2:**
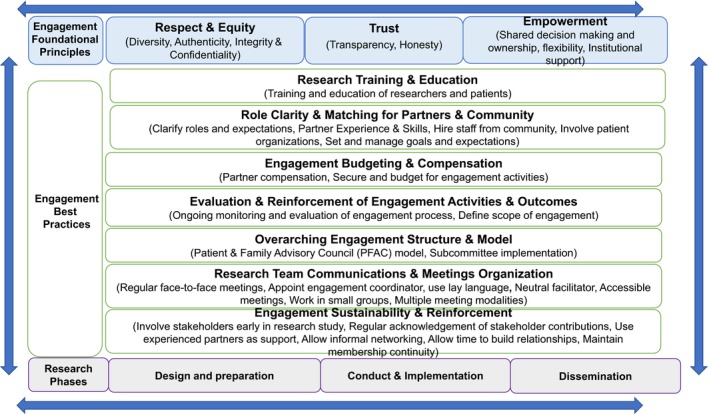
Foundational framework summarizing principles and best practice activities supporting patient stakeholder engagement in research

An overarching foundational framework summarizing the key findings from this narrative review is shown in Figure 2. In this framework, three distinct but inter‐related elements of patient stakeholder engagement in research are conceptualized namely “foundational principles,” “best practices” and “research phases.” Within the “foundational principles,” element are thematically grouped foundational principles from Table 1. Similarly, within the “best practices,” element are thematically grouped activities from Table 2. The final element of the framework describes the three distinct “research phases” of study where engagement should occur—design and preparation, conduct and implementation and dissemination. All elements within this framework are inter‐related as shown by the circular arrows surrounding them. For example, the foundational engagement principles should infuse all best practices, which are subsequently relevant to activities within each research phase.

## 4 DISCUSSION

Engaging patient stakeholders in research in partnership as advisors, collaborators and co‐investigators presents an opportunity to design, implement and disseminate patient‐centred research. These partnerships can better ensure that research incorporates the patient stakeholder voice including their priorities and preferences. Engagement efforts facilitate patient‐centred research design, implementation and dissemination. Our review of a selection of publicly available repository of systematically identified literature^26^ for the first time summarizes existing frameworks and commentary to quantify the key foundational principles of patient stakeholder engagement as well as best practice recommendations that can assist operationalize these partnerships during the life cycle of a research project. Our review also creates an overarching foundational framework that summarizes guidance for authentic and sustainable partnerships between patient stakeholders and researchers.

The most commonly reported foundational elements of patient stakeholder engagement are respect, equitable power and trust. This is not surprising as these elements are consistently highlighted in qualitative studies exploring patient stakeholder experiences of engaging in research. For example, telephone interviews with patient stakeholders in the United States found that the relationships with researchers had to be respectful and trusting for them to be a success.^56^ Commentaries by patient stakeholders note that mutual trust was key to keeping open dialogue flowing with the research team.^24^, ^53^, ^75^, ^76^ A number of focus group studies have reported that equitable and shared collaborative decision making is essential during the research engagement process.^19^, ^59^

A number of articles identified in this review note the importance of acknowledging and addressing the diversity of patient stakeholders who are engaged as partners in research, a well‐established challenge.^56^, ^77^, ^78^ Addressing diversity through efforts that are now emerging to help overcome the challenges^77^, ^79^ can better ensure a range of voices are captured that will enhance the generalizability of the findings. One key suggestion is that researchers conduct outreach and bring research to the communities where people live.^77^, ^79^ Further, appreciating that some groups may be apprehensive to partner with research teams due to historical mistrust must also be considered when partnering with certain populations.^77^ This means during engagement that researchers should focus on creating mutual respect, trust and openness—key foundational principles of engagement identified in this review.

Authors identified in this review proposed that the most effective model for patient stakeholder engagement is that of Patient and Family Advisory Councils (PFACs). These councils support patient stakeholders to meet regularly at a health‐care institution and share their experiences of care or collective perspectives on a specific topic.^80^ Harnessing these existing structures is advantageous for research given PFAC members are already orientated and engaged.^23^ However, using the PFAC model is only one approach. Other opportunities exist that may allow for greater outreach and an increase in the diversity of engaged stakeholders, without necessarily requiring in‐person meetings: for instance, the use of e‐advisors^81^ and online communities such as the “Patients Like Me” collaborative that has informed type II diabetes research.^82^

The summary framework derived from our review also describes a range of best practice activities, or actions, that build on the foundational elements of patient stakeholder engagement in research. The most frequently noted activity to support engagement was training for both patient stakeholders and researchers. While training is not intended to create researchers of patient stakeholders, which could mean they lose their unique perspective, orientation to research processes, to research topics and to working within a research team has been shown to enhance their participation and engagement. Equally as necessary is researcher training in how to authentically and effectively engage patient stakeholders—a skill deficiency cited by researchers.^17^, ^20^ Co‐learning of patient stakeholders and researchers has also been described as an opportunity to further enhance the relationship between all members of the research team. A number of education and training resources are freely available to support this process.^13^, ^83^, ^84^, ^85^, ^86^, ^87^

Compensation for patient stakeholders for their expertise and time, and reimbursement for out‐of‐pocket expenses was cited as good practice in articles identified in this review. While these practices are encouraged by funding agencies and are dependent on the level of engagement,^11^, ^88^ both remain a challenge to operationalize in practice given they are often not budgeted for. Stipends or reimbursements for out‐of‐pocket expenses should be viewed as an absolute minimum. While the motivation of patient stakeholders to engage and participate in research is altruistic,^7^ it is imperative that if they contribute during the life cycle of a research project, compensation should be equitable, transparent or even customized to the individual patient stakeholder needs.^30^

Another activity noted in our review was to regularly evaluate the engagement process during the life cycle of a research project. This allows for engagement efforts to be recalibrated based on feedback and ensures patient stakeholders remain active and informed partners. While there have been significant efforts to describe the benefits, or value, of engaging patient stakeholders in research such as improved research quality, processes, recruitment and retention rates,^3^, ^4^, ^6^, ^10^ there are currently no validated tools that can evaluate the actual process of research engagement, or the experiences of patient stakeholders who are engaged. Our review has identified potential domains (Tables 1 and 2) that could be used to inform items in such an evaluation tool. Similarly, Hamilton and colleague's conceptual framework for patient engagement in research describes eight relevant themes that could also be used as a basis for evaluation.^89^ There is an urgent need to develop an evaluation tool to ensure that the engagement process is meaningful, authentic and a positive experience for both patient stakeholders and researchers.^16^

Our review has a number of limitations including the potential for selection bias given that only English language bibliographic databases were searched, meaning some relevant articles would have been missed. Other English language articles may also have not been included in this review due to the poorly standardized taxonomy and nomenclature related to patient stakeholder engagement in research. Given this limitation, we strongly recommend that new Medical Subject Headings (MeSH) terms are submitted to the National Library that would capture patient engagement in the peer‐reviewed literature. Terms such as “patient engagement,” “patient involvement,” or “patient‐centered outcomes research,” or “patient partner,” and other relevant terms would be extremely helpful for identifying such literature. Further, we acknowledge that the search terms used to identify articles in this review are US centric and do not include terminology used in some international settings. However, given the broad search terms used to create the repository accessed for this review, the number of articles missed would likely be small. It is also essential to apply some caution to our results in that just because a foundational principle, or best practice, is not frequency reported this does not imply that it is less important or applicable. The science of patient stakeholder engagement in research is new and evolving meaning we do not know yet which principles or practices are most important.

In summary, this narrative review has summarized the foundational principles of engagement between researchers and patient stakeholders and describes best practice activities to support this process. This information can be used to facilitate patient‐centred research, thereby ensuring the patient stakeholder voice and perspective remains central.

## CONFLICTS OF INTEREST

The authors have no conflicts of interest to declare.

## References

[1] Pomey M , Hihat H , Khalifa M , Lebel P . Patient partnerships in quality improve. Patient Exp J. 2015;2:29‐42.

[2] Amirav I , Vandall‐Walker V , Rasiah J , Saunders L . Patient and researcher engagement in health research: A parent’s perspective. Pediatrics. 2017;140(3):e20164127. 28851740 10.1542/peds.2016-4127

[3] Dudley L , Gamble C , Preston J , et al. What difference does patient and public involvement make and what are its pathways to impact? Qualitative study of patients and researchers from a cohort of randomised clinical trials. PLoS ONE. 2015;10(6):e0128817. 26053063 10.1371/journal.pone.0128817 PMC4459695

[4] Domecq JP , Prutsky G , Elraiyah T , et al. Patient engagement in research: a systematic review. BMC Health Serv Res. 2014;14:89. 24568690 10.1186/1472-6963-14-89 PMC3938901

[5] Sheridan S , Schrandt S , Forsythe L , Hilliard TS , Paez KA . The PCORI engagement rubric: promising practices for partnering in research. Ann Fam Med. 2017;15(2):165‐170. 28289118 10.1370/afm.2042 PMC5348236

[6] Brett J , Staniszewska S , Mockford C , et al. Mapping the impact of patient and public involvement on health and social care research: a systematic review. Heal Expect. 2014;17(5):637‐650. 10.1111/j.1369-7625.2012.00795.x PMC5060910 22809132

[7] Zelmer J . Engaging citizens and patients in health research: learning from experience. Healthc Policy. 2016;12(1):8‐11. PMC5008125 27585021

[8] Bombak A , Hanson H . A critical discussion of patient engagement in research. J Patient Cent Res Rev. 2017;4:39‐41. 10.17294/2330-0698.1273 PMC6664365 31413969

[9] Lavallee DC , Williams CJ , Tambor ES , Deverka PA . Stakeholder engagement in comparative effectiveness research: how will we measure success? J Comp Eff Res. 2012;1(5):397‐407. 24236417 10.2217/cer.12.44

[10] INVOLVE . Exploring the impact of public involvement on the quality of Research. Eastleigh, 2013.

[11] Sheridan S , Schrandt S .A framework for financial compensation for patient partners in research. https://www.pcori.org/blog/framework-financial-compensation-patient-partners-research. Accessed August 5, 2018.

[12] Esmail L , Moore E , Rein A . Evaluating patient and stakeholder engagement in research: moving from theory to practice. J Comp Eff Res. 2015;4(2):133‐145. 25825842 10.2217/cer.14.79

[13] Briefing I . Notes for researchers: public involvement in NHS. Public Health and Social Care Research. Eastleigh. 2012.

[14] Canadian Institutes of Health Research . Strategy for Patient‐Orientated Research (SPOR): Patient Engagement Framework. Ottawa, ON: Canadian Institutes of Health Research, 2014.

[15] Forsythe L , Heckert A , Margolis MK , Schrandt S , Frank L . Methods and impact of engagement in research, from theory to practice and back again: early findings from the Patient‐Centered Outcomes Research Institute. Qual Life Res. 2018;27(1):17‐31. 28500572 10.1007/s11136-017-1581-x PMC5770504

[16] Manafo E , Petermann L , Mason‐Lai P , Vandall‐Walker V . Patient engagement in Canada: a scoping review of the ‘how’ and ‘what’ of patient engagement in health research. Heal Res Policy Syst. 2018;16:5. 10.1186/s12961-018-0282-4 PMC5804082 29415734

[17] Forsythe LP , Ellis LE , Edmundson L , et al. Patient and stakeholder engagement in the PCORI Pilot Projects: Description and lessons learned. J Gen Intern Med. 2016;31(1):13‐21. 26160480 10.1007/s11606-015-3450-z PMC4700002

[18] Manafò E , Petermann L , Vandall‐Walker V , Mason‐Lai P . Patient and public engagement in priority setting: A systematic rapid review of the literature. PLoS ONE. 2018;13(3):e0193579. 29499043 10.1371/journal.pone.0193579 PMC5834195

[19] Harrison JD , Anderson WG , Fagan M , et al. Patient and Family Advisory Councils (PFACs): identifying challenges and solutions to support engagement in research. Patient Centered Outcomes Res. 2018;11(4):413‐423. 10.1007/s40271-018-0298-4 PMC11034744 29392529

[20] Motu'apuaka M , Whitlock E , Kato E , et al. Defining the benefits and challenges of stakeholder engagement in systematic reviews. Comp Eff Res. 2015;5:13.

[21] Hahn D , Hoffmann A , Felzien M , LeMaster J , Xu J , Fagnan L . Tokenism in Patient Engagement. Fam Pract 2016;34: cmw09. 10.1093/fampra/cmw097 27660557

[22] Frank L , Forsythe L , Ellis L , et al. Conceptual and practical foundations of patient engagement in research at the patient‐centered outcomes research institute. Qual Life Res. 2015;24(5):1033‐1041. 25560774 10.1007/s11136-014-0893-3 PMC4412554

[23] Fagan MB , Morrison CR , Wong C , Carnie MB , Gabbai‐Saldate P . Implementing a pragmatic framework for authentic patient‐researcher partnerships in clinical research. J Comp Eff Res. 2016;5(3):297‐308. 27144508 10.2217/cer-2015-0023

[24] Hewlett S , de Wit M , Richards P , et al. Patients and professionals as research partners: challenges, practicalities, and benefits. Arthritis Rheum. 2006;55(4):676‐680. 16874772 10.1002/art.22091

[25] Moher D , Liberati A , Tetzlaff J , Altman DG . Preferred reporting items for systematic reviews and meta‐analyses: the PRISMA statement. J Clin Epidemiol. 2009;62(10):1006‐1012. 19631508 10.1016/j.jclinepi.2009.06.005

[26] Patient Centered Outcomes Research Institute (PCORI). Engagement in Health Research Literature Explorer. https://www.pcori.org/literature/engagement-literature. Accessed August 4, 2018.

[27] Bradley EH , Curry LA , Devers KJ . Qualitative data analysis for health services research: developing taxonomy, themes, and theory. Health Serv Res. 2007;42(4):1758‐1772. 17286625 10.1111/j.1475-6773.2006.00684.x PMC1955280

[28] Kirwan JR , de Wit M , Frank L , et al. Emerging guidelines for patient engagement in research. Value Heal J Int Soc Pharmacoeconomics Outcomes Res. 2017;20(3):481‐486. 10.1016/j.jval.2016.10.003 28292494

[29] Ahmed SM , Palermo A‐GS . Community engagement in research: frameworks for education and peer review. Am J Public Health. 2010;100(8):1380‐1387. 20558798 10.2105/AJPH.2009.178137 PMC2901283

[30] Arkind J , Likumahuwa‐Ackman S , Warren N , et al. Lessons learned from developing a patient engagement panel: an OCHIN report. J Am Board Fam Med. 2015;28(5):632‐638. 26355135 10.3122/jabfm.2015.05.150009 PMC4904782

[31] Bagley HJ , Short H , Harman NL , et al. A patient and public involvement (PPI) toolkit for meaningful and flexible involvement in clinical trials—a work in progress. Res Involv Engagem. 2016;2:15. 29062516 10.1186/s40900-016-0029-8 PMC5611579

[32] Baquet CR . A model for bidirectional community‐academic engagement (CAE): overview of partnered research, capacity enhancement, systems transformation, and public trust in research. J Health Care Poor Underserved. 2012;23(4):1806‐1824. 23698691 10.1353/hpu.2012.0155 PMC5393451

[33] Boote J , Telford R , Cooper C . Consumer involvement in health research: a review and research agenda. Health Policy. 2002;61(2):213‐236. 12088893 10.1016/s0168-8510(01)00214-7

[34] Buck D , Gamble C , Dudley L , et al. From plans to actions in patient and public involvement: qualitative study of documented plans and the accounts of researchers and patients sampled from a cohort of clinical trials. BMJ Open. 2014;4(12):e006400. 10.1136/bmjopen-2014-006400 PMC4256646 25475243

[35] Cargo M , Mercer SL . The value and challenges of participatory research: strengthening its practice. Annu Rev Public Health. 2008;29:325‐350. 18173388 10.1146/annurev.publhealth.29.091307.083824

[36] Carman KL , Dardess P , Maurer M , et al. Patient and family engagement: a framework for understanding the elements and developing interventions and policies. Health Aff (Millwood). 2013;32(2):223‐231. 23381514 10.1377/hlthaff.2012.1133

[37] Concannon TW , Meissner P , Grunbaum JA , et al. A new taxonomy for stakeholder engagement in patient‐centered outcomes research. J Gen Intern Med. 2012;27(8):985‐991. 22528615 10.1007/s11606-012-2037-1 PMC3403141

[38] de Wit M , Elberse JE , Broerse J , Abma TA . Do not forget the professional–the value of the FIRST model for guiding the structural involvement of patients in rheumatology research. Heal Expect an Int J public Particip Heal care Heal policy. 2015;18(4):489‐503. 10.1111/hex.12048 PMC5060802 23363240

[39] de Wit M , Kvien TK , Gossec L . Patient participation as an integral part of patient‐reported outcomes development ensures the representation of the patient voice: a case study from the field of rheumatology. RMD Open. 2015;1(1):e000129. 26509075 10.1136/rmdopen-2015-000129 PMC4613173

[40] Deverka PA , Lavallee DC , Desai PJ , et al. Stakeholder participation in comparative effectiveness research: defining a framework for effective engagement. J Comp Eff Res. 2012;1(2):181‐194. 22707880 10.2217/cer.12.7 PMC3371639

[41] Dewar BJ . Beyond tokenistic involvement of older people in research ‐ a framework for future development and understanding. J Clin Nurs. 2005;14(Suppl 1):48‐53. 15819660 10.1111/j.1365-2702.2005.01162.x

[42] Duffett L . Patient engagement: What partnering with patient in research is all about. Thromb Res. 2017;150:113‐120. 27817863 10.1016/j.thromres.2016.10.029

[43] Fairbrother P , McCloughan L , Adam G , et al. Involving patients in clinical research: the Telescot Patient Panel. Heal Expect. 2016;19(3):691‐701. 10.1111/hex.12132 PMC5055252 24112277

[44] Forsythe LP , Szydlowski V , Murad MH , et al. A systematic review of approaches for engaging patients for research on rare diseases. J Gen Intern Med. 2014;29(Suppl 3):S788‐800. 25047393 10.1007/s11606-014-2895-9 PMC4124116

[45] Guise J‐M , O’Haire C , McPheeters M , et al. A practice‐based tool for engaging stakeholders in future research: a synthesis of current practices. J Clin Epidemiol. 2013;66(6):666‐674. 23497857 10.1016/j.jclinepi.2012.12.010

[46] Hoffman A , Montgomery R , Aubry W , Tunis SR . How best to engage patients, doctors, and other stakeholders in designing comparative effectiveness studies. Health Aff (Millwood). 2010;29(10):1834‐1841. 20921483 10.1377/hlthaff.2010.0675

[47] INVOLVE . Principles and Standards for Public Involvement in Research. Eastleigh, 2013.

[48] INVOLVE . Public Involvement in Research: Values and Principles Framework. Eastleigh, 2015.

[49] Israel BA , Krieger J , Vlahov D , et al. Challenges and facilitating factors in sustaining community‐based participatory research partnerships: lessons learned from the Detroit, New York City and Seattle Urban Research Centers. J Urban Health. 2006;83(6):1022‐1040. 17139552 10.1007/s11524-006-9110-1 PMC3261295

[50] James S , Arniella G , Bickell NA , et al. Community ACTION boards: an innovative model for effective community‐academic research partnerships. Prog Community Health Partnersh. 2011;5(4):399‐404. 22616207 PMC3437746

[51] Jenner MK , Gilchrist M , Baker GC . Practical considerations in improving research through public involvement. Res Involv Engagem. 2015;1:3. 29062492 10.1186/s40900-015-0002-y PMC5598088

[52] Jinks C , Carter P , Rhodes C , et al. Patient and public involvement in primary care research ‐ an example of ensuring its sustainability. Res Involv Engagem. 2016;2(1):1. 29062502 10.1186/s40900-016-0015-1 PMC5611572

[53] Johnson DS , Bush MT , Brandzel S , Wernli KJ . The patient voice in research‐evolution of a role. Res Involv Engagem. 2016;2:6. 29062507 10.1186/s40900-016-0020-4 PMC5611646

[54] Langston AL , McCallum M , Campbell MK , Robertson C , Ralston SH . An integrated approach to consumer representation and involvement in a multicentre randomized controlled trial. Clin Trials. 2005;2(1):80‐87. 16279582 10.1191/1740774505cn065oa

[55] Lavallee DC , Wicks P , Alfonso Cristancho R , Mullins CD . Stakeholder engagement in patient‐centered outcomes research: high‐touch or high‐tech? Expert Rev Pharmacoecon Outcomes Res. 2014;14(3):335‐344. 24661181 10.1586/14737167.2014.901890

[56] Lavallee D , Gore J , Lawrence S , et al.Initiative to Support Patient Involvement in Research (INSPIRE). https://www.becertain.org/sites/default/files/INSPIREPhaseIReportFinal2016.09.30.pdf, 2016. Accessed August 4, 2018.

[57] Lindenmeyer A , Hearnshaw H , Sturt J , Ormerod R , Aitchison G . Assessment of the benefits of user involvement in health research from the Warwick Diabetes Care Research User Group: a qualitative case study. Heal Expect an Int J public Particip Heal care Heal policy. 2007;10(3):268‐277. 10.1111/j.1369-7625.2007.00451.x PMC5060408 17678515

[58] Madrid S , Tuzzio L , Stults C , et al. Sharing experiences and expertise: the Health Care Systems Research Network workshop on patient engagement in research. J Patient Cent Res Rev. 2016;3:159‐166.

[59] Marlett N , Shklarov S , Marshall D , Santana MJ , Wasylak T . Building new roles and relationships in research: a model of patient engagement research. Qual Life Res. 2015;24(5):1057‐1067. 25377348 10.1007/s11136-014-0845-y

[60] Marsden J , Bradburn J . Patient and clinician collaboration in the design of a national randomized breast cancer trial. Heal Expect. 2004;7(1):6‐17. 10.1111/j.1369-7625.2004.00232.x PMC5060213 14982495

[61] McAllister CL , Green BL , Terry MA , Herman V , Mulvey L . Parents, practitioners, and researchers: community‐based participatory research with early head start. Am J Public Health. 2003;93(10):1672‐1679. 14534219 10.2105/ajph.93.10.1672 PMC1448031

[62] McNeil H , Elliott J , Huson K , et al. Engaging older adults in healthcare research and planning: a realist synthesis. Res Involv Engagem. 2016;2:10. 29062511 10.1186/s40900-016-0022-2 PMC5611557

[63] Newman SD , Andrews JO , Magwood GS , Jenkins C , Cox MJ , Williamson DC . Community advisory boards in community‐based participatory research: a synthesis of best processes. Prev Chronic Dis. 2011;8(3):A70. 21477510 PMC3103575

[64] Nierse CJ , Schipper K , van Zadelhoff E , van de Griendt J , Abma TA . Collaboration and co‐ownership in research: dynamics and dialogues between patient research partners and professional researchers in a research team. Heal Expect an Int J public Particip Heal care Heal policy. 2012;15(3):242‐254. 10.1111/j.1369-7625.2011.00661.x PMC5060620 21332617

[65] Pandi‐Perumal SR , Akhter S , Zizi F , et al. Project stakeholder management in the clinical research environment: how to do it right. Front psychiatry. 2015;6:71. 26042053 10.3389/fpsyt.2015.00071 PMC4434843

[66] Pandya‐Wood R , Barron DS , Elliott J . A framework for public involvement at the design stage of NHS health and social care research: time to develop ethically conscious standards. Res Involv Engagem. 2017;3:6. 29062531 10.1186/s40900-017-0058-y PMC5611655

[67] Perlmutter J , Roach N , Lou SM . Involving advocates in cancer. Research. Semin Oncol. 2015;42(5):681‐685. 26433549 10.1053/j.seminoncol.2015.07.008

[68] Rhodes P , Nocon A , Booth M , et al. A service users’ research advisory group from the perspectives of both service users and researchers. Health Soc Care Community. 2002;10(5):402‐409. 12390226 10.1046/j.1365-2524.2002.00376.x

[69] Salsberg J , Parry D , Pluye P , Macridis S , Herbert CP , Macaulay AC . Successful strategies to engage research partners for translating evidence into action in community health: a critical review. J Environ Public Health. 2015;2015:191856. 25815016 10.1155/2015/191856 PMC4359847

[70] Shalowitz MU , Isacco A , Barquin N , et al. Community‐based participatory research: a review of the literature with strategies for community engagement. J Dev Behav Pediatr. 2009;30(4):350‐361. 19672162 10.1097/DBP.0b013e3181b0ef14

[71] Shippee ND , Domecq Garces JP , Prutsky Lopez GJ , et al. Patient and service user engagement in research: a systematic review and synthesized framework. Heal Expect. 2015;18(5):1151‐1166. 10.1111/hex.12090 PMC5060820 23731468

[72] Suarez‐Balcazar Y , Harper GW , Lewis R . An interactive and contextual model of community‐university collaborations for research and action. Heal Educ Behav. 2005;32(1):84‐101. 10.1177/1090198104269512 15642756

[73] Supple D , Roberts A , Hudson V , et al. From tokenism to meaningful engagement: best practices in patient involvement in an EU project. Res Involv Engagem. 2015;1:5. 29062494 10.1186/s40900-015-0004-9 PMC5598090

[74] Abma TA , Broerse J . Patient participation as dialogue: setting research agendas. Heal Expect. 2010;13(2):160‐173. 10.1111/j.1369-7625.2009.00549.x PMC5060528 20536537

[75] Robbins M , Tufte J , Hsu C . Learning to “Swim” with the experts: experiences of two patient co‐investigators for a project funded by the patient‐centered outcomes research institute. Perm J. 2016;20(2):85‐88. 10.7812/TPP/15-162 PMC4867832 27083011

[76] Tai‐Seale M , Sullivan G , Cheney A , Thomas K , Frosch D . The language of engagement: “Aha!” moments from engaging patients and community partners in two pilot projects of the patient‐centered outcomes research institute. Perm J. 2016;20(2):89‐92. 26909777 10.7812/TPP/15-123 PMC4867833

[77] Kauffman KS , Dosreis S , Ross M , Barnet B , Onukwugha E , Mullins CD . Engaging hard‐to‐reach patients in patient‐centered outcomes research. J Comp Eff Res. 2013;2(3):313‐324. 24236630 10.2217/cer.13.11

[78] Sofolahan‐Oladeinde Y , Newhouse RP , Lavallee DC , Huang JC , Mullins CD . Early assessment of the 10‐step patient engagement framework for patient‐centred outcomes research studies: the first three steps. Fam Pract. 2017;34(3):272‐277. 28334775 10.1093/fampra/cmx013

[79] Harrison JD , Anderson WG , Fagan M , et al.Patient & Family Advisory Councils (PFACs): Recruiting and supporting members from diverse, vulnerable and under‐represented communities. Journal of Hospital Medicine. https://www.shmabstracts.com/abstract/patient-family-advisory-councils-pfacs-recruiting-and-supporting-members-from-diverse-vulnerable-and-under-represented-communities/. Accessed August 4, 2018.

[80] Fagan M , Wong C , Morrison C , Lewis‐O’Connor A , Carnie M . Patients, persistence and partnerships: Creating and sustaining patient and family advisory councils in a hospital setting. JCOM. 2016;23:219‐225.

[81] Vanderbilt Health .Advise Vanderbilt. 2018; https://www.advisevanderbilt.com/Portal/default.aspx

[82] Simacek KF , Nelson T , Miller‐Baldi M , Bolge SC . Patient engagement in type 2 diabetes mellitus research: what patients want. Patient Prefer Adherence. 2018;12:595‐606. 29720875 10.2147/PPA.S159707 PMC5918623

[83] CERTAIN Patient Advisory Network’s INSPIRE Research Portal. http://inspireresearch.org/home. Accessed August 4, 2018.

[84] Institute for Patient and Family Centered Care . A toolbox for creating sustainable partnerships with patients and families in research. http://www.ipfcc.org/bestpractices/sustainable-partnerships/index.html. Accessed August 4, 2018.

[85] National Health Service England . Bite size guides to participation. https://www.england.nhs.uk/publication/bite-size-guides-to-participation/. Accessed August 4, 2018.

[86] Agency for Healthcare Quality & Research . Guide to patient and family engagement in hospital quality and safety. https://www.ahrq.gov/professionals/systems/hospital/engagingfamilies/guide.html. Accessed August 4, 2018.

[87] Alberta INNOVATES . Patient Engagement Resources. 2018; https://albertainnovates.ca/our-health-innovation-focus/the-alberta-spor-support-unit/patient-engagement-platform/resources/

[88] INVOLVE . Policy on payment of fees and expenses for members of the public actively involved with INVOLVE. Eastleigh, 2012.

[89] Hamilton CB , Hoens AM , Backman CL , et al. An empirically based conceptual framework for fostering meaningful patient engagement in research. Heal Expect. 2018;21(1):396‐406. 10.1111/hex.12635 PMC5750689 28984405

