# Supplementary material for: Patient stakeholder engagement in research: A narrative review to describe foundational principles and best practice activities
Source: Health Expect. 2019 Feb 13;22(3):307–16. doi: 10.1111/hex.12873 (PMC6543160; doi:10.1111/hex.12873)
Supplement: Supplementary file 1 [file HEX-22-307-s001.docx]

**Appendix 1A: Bibliographic literature database search strategy**

Patient Participation [Mesh] OR patient input [tw] OR patient collaboration [tw] OR engaging patients [tw] OR engages patients [tw] OR engaged patients [tw] OR patient partner [tw] OR patient partnership [tw] OR patient partners [tw] OR patient participation [tw] OR stakeholder participation [tw] OR engages stakeholder [tw] OR engage stakeholders [tw] OR engaged stakeholders [tw] OR engaging stakeholders [tw] OR stakeholder partnership [tw] OR stakeholder partners [tw] OR consumer participation [tw] OR community participation [tw] OR public participation [tw] OR patient engagement [tw] OR stakeholder engagement [tw] OR consumer engagement [tw] OR community engagement [tw] OR public engagement [tw] OR patient involvement [tw] OR stakeholder involvement [tw] OR consumer engagement [tw] OR community involvement [tw] OR public involvement [tw] OR public partners [tw] OR public partnerships [tw] OR lay participation [tw] OR lay engagement [tw] OR lay involvement [tw] OR OR engaging community [tw] OR engaged community [tw] OR engaging communities [tw] OR engaged communities [tw] OR community partner [tw] OR community partnership [tw] OR community partners [tw] OR payer engagement [tw] OR payer involvement [tw] OR insurance participation [tw] OR employer involvement [tw] OR employer participation [tw] OR employer engagement [tw] OR pharmaceutical involvement [tw] OR engaging caregivers [tw] OR engaged caregivers [tw] OR caregiver engagement [tw] OR caregiver involvement [tw] OR caregiver participation [tw] OR caregiver partner [tw] OR consumer collaboration [tw] OR engaging consumers [tw] OR engaged consumers [tw] PR consumer partnerships [tw] OR provider engagement [tw] OR provider participation [tw] OR provider involvement [tw] OR clinician engagement [tw] OR clinician involvement [tw] OR clinician participation [tw] OR engaged research [tw] OR engagement research [tw] OR OR research engagement [tw] OR engagement framework [tw] OR facilitating engagement [tw] OR patient and public involvement [tw] OR partnered research [tw] OR participatory research [tw] OR participatory action research [tw] OR community-academic partnership [tw] OR community-engaged research [tw] OR engagement method [tw] OR public involvement impact assessment framework [tw] NOT preference [tw] AND comparative effectiveness research [tw] OR CER [tw] OR randomized controlled trial [tw] OR randomized clinical trial [tw] OR randomized clinical trial [tw] OR randomized controlled trial [tw] OR RCT [tw] OR clinical trial [tw] OR clinical control trial [tw] OR clinical controlled trial [tw] OR observational study [tw] OR comparative study [tw] OR pragmatic trial [tw] OR pragmatic clinical trial [tw] OR meta analysis [tw] OR systematic review [tw] OR comparative effectiveness trial [tw] OR clinical trial [ptyp] OR comparative study [ptyp] OR meta-analysis [ptyp] OR observational study [ptyp] OR pragmatic clinical trial [ptyp] OR randomized controlled trial [ptyp] OR editorial [ptyp] OR letter [ptyp] OR comment [ptyp] OR conceptual model [tiab] OR practical model [tiab] OR conceptual framework [tiab] OR practical framework [tiab] OR systematic [sb] OR evaluation studies [ptyp] AND English [lang]

**Appendix 1B; Article repository inclusion and exclusion criteria**

| **Inclusion Criteria** | **Exclusion Criteria** |
| --- | --- |
| - English  - Health research (NOT limited to exclusively comparative effectiveness research (CER))  - Article addresses engagement in research (e.g. planning, conducting or disseminating research)  - For bucket 1: manuscripts focused on primary health-related research question and additionally report observations about the about the impact or effects or patient stakeholder engagement in their research findings  - For bucket 2: manuscripts focused on a primary engagement question within the context of health-related research question with reporting of engagement methodologies and impact  - For bucket 3: formal qualitative, quantitative or mixed methods evaluation of methods for engagement, the impact of engagement or another research objective for the study of research engagement  - For bucket 4: scientific commentaries, opinion pieces and conceptual frameworks that provide models or frameworks for engagement, examples of engagement, discussing the impact of engagement or providing otherwise relevant commentary | **Exclusion at abstract screening:**  Articles excluded for the reasons below would not be forwarded for full-text screening, unless additional information was needed.  - Duplicates  - Not in English  - Not in health research (e.g. agricultural, occupational health, environmental health)  - Studies evaluating engagement in health care rather than engagement in research  - Patient preference studies (health care preferences) (e.g. research exclusively asking what do patients prefer or want in their care)  - Patient stakeholder administering is the ONLY role in the task. No other form of patient stakeholder engagement in the research task  **Exclusion at full-text screening:**  In some cases, an article was forwarded to the full-text screening because more information was needed to make a final determination about inclusion in the repository. Articles were excluded at the full text screening phase if they met the following criteria:  - For buckets 1 and 2: No description of the effects of engagement in research (i.e. study only mentions they engaged patients in research without describing at a minimum, how learnings from partners were incorporated, changes made as a result of engagement, other perceived impact of engagement  - Protocols that describe how stakeholders will be engaged in the future, instead of reflections or evaluations of past engagement  - Studies focused on stakeholder involvement only for recruitment of study participants  Studies focusing on stakeholder involvement in developing clinical guidelines, rather than developing the research process/content itself  New articles were introduced for screening after the title/abstract screening process had already been completed. At this point in the full-text screening process articles could be excluded from the repository for any of the above listed criteria. |
