# Supplementary material for: Patient stakeholder engagement in research: A narrative review to describe foundational principles and best practice activities
Source: Health Expect. 2019 Feb 13;22(3):307–16. doi: 10.1111/hex.12873 (PMC6543160; doi:10.1111/hex.12873)
Supplement: Supplementary file 2 [file HEX-22-307-s002.docx]

**Frameworks, toolkits, guidelines supporting patient engagement in research review:**

**Publication name:**

**Authors:**

**Year published:**

**Country of origin**:

**Type of Support**

Educational Toolkit ❑ General Engagement Guidelines ❑

Framework ❑ Other _______________________________________________

________________________________________________

**1. Development of educational toolkit, framework or general engagement guidelines**

| **Theoretical basis (describe):** |
| --- |
| **Setting where developed:** |
| **Target Participants:** |
| Patient ❑ Caregiver ❑  Family ❑ Researcher ❑ |

Other _______________________________________________

| **Target participants setting**: |
| --- |
| \| PFAC ❑ General patient Stakeholder Population ❑  Disease specific ❑ Researcher ❑ \| \| --- \|   Other _______________________________________________ |

| **Comments on developing process:** |
| --- |

**2. Components/domains/recommendations of toolkit, framework or guideline**

**Describe details:**

**3. Toolkit, framework or guidelines use**

**Is instrument being used in practice**? No ❑ Yes ❑

| **If yes provide details:** |
| --- |
|  |
|  |
|  |

**4. Has the toolkit/framework/guideline been evaluated?**

| **Details:** |
| --- |
|  |
|  |
|  |

**5. Other relevant comments:**

|  |
| --- |
|  |
|  |
|  |

**5. Relevant references:**

**Appendix 2**
