# Supplementary material for: Patient stakeholder engagement in research: A narrative review to describe foundational principles and best practice activities
Source: Health Expect. 2019 Feb 13;22(3):307–16. doi: 10.1111/hex.12873 (PMC6543160; doi:10.1111/hex.12873)
Supplement: Supplementary file 3 [file HEX-22-307-s003.docx]

**Appendix 3: Articles Identified in Narrative Review Describing Foundation Principles and Best Practices for Patient Stakeholder Engagement in Research**

| **AUTHOR** | **TITLE** | **YEAR** | **COUNTRY OF ORIGIN** | **SUMMARY** |
| --- | --- | --- | --- | --- |
| Abma et al | Patient participation as dialogue: setting research agendas. | 2010 | The Netherlands | Authors use seven case studies between 2003 and 2007 to develop and validate a Dialogue Model for patient participation in health research agenda setting, emphasizing that participation is in principle a dialogical process between stakeholders, including patients, and that mutual learning between stakeholders occurs by means of ongoing dialogues. |
| Ahmed et al | Community Engagement in Research: Frameworks for Education and Peer Review. | 2010 | US | A community engagement framework created by The National Institutes of Health [NIH] Director’s Council of Public Representatives in order to promote authentic community-academic partnerships. It defines values, strategies to operationalize each value, and potential outcomes of their use, as well as a peer-review framework for evaluating research that engages communities. |
| Arkind et al | Lessons Learned from Developing a Patient Engagement Panel: An OCHIN Report | 2015 | US | Describes early lessons learned from the development of OCHIN’s Patient Engagement Panel (PEP). |
| Bagley et al | A patient and public involvement (PPI) toolkit for meaningful and flexible involvement in clinical trials – a work in progress | 2016 | UK | Details the initial phases of the development of a web-based toolkit designed for use by chief investigators and study teams to facilitate meaningful and effective patient and public involvement [PPI] at all stages of a clinical trial. |
| Baquet et al | A Model for Bidirectional Community-Academic Engagement (CAE): Overview of Partnered Research, Capacity Enhancement, Systems Transformation, and Public Trust in Research. | 2012 | US | Describes the planning and implementation of The University of Maryland’s Office of Policy and Planning model for community-academic engagement [CAE] in partnered research and programs. |
| Boote et al | Consumer involvement in health research: a review and research agenda. | 2001 | UK | Authors set out to answer several questions through literature review: 1) how can consumer involvement in health care be conceptualized? 2) how and why does consumer involvement influence research? 3) how can that influence be measured/evaluated? 4) what factors are associated with “successful” consumer involvement in health research? |
| Buck et al | From plans to actions in patient and public involvement: qualitative study of documented plans and the accounts of researchers and patients sampled from a cohort of clinical trials. | 2014 | UK | Examines proposed and actual methods/plans for patient and public involvement (PPI) in research across 28 trials, compares grant applications to patients’ and researchers’ reflections of the process via interview, then addresses established challenges and best practices. |
| Canadian Institutes of Health Research | Strategy for Patient Orientated Research (SPOR): Patient Engagement Framework | 2014 | Canada | Reports findings of a workshop that involved cross-representation of patients and patient engagement experts. At this workshop key concepts, principles and areas for patient engagement were defined. |
| Cargo et al | The Value and Challenges of Participatory Research: Strengthening Its Practice. | 2008 | Canada, Australia, US | A critical review of the participatory research [PR] literature, culminating in the development of an integrative practice framework that provides a structured process for developing and maintaining PR partnerships, designing and implementing PR efforts, and evaluating outcomes of PR studies. |
| Carman et al | Patient and Family Engagement: A Framework For Understanding The Elements And Developing Interventions And Policies. | 2013 | US | Discusses the levels at which patient engagement can occur across the health care system and explores the factors that influence whether and to what extent engagement occurs. |
| Concannon et al | A New Taxonomy for Stakeholder Engagement in Patient-Centered Outcomes Research. | 2012 | US | Authors introduce a flexible taxonomy called the 7Ps of Stakeholder Engagement and Six Stages of Research for identifying stakeholders for patient-centered outcomes research and developing engagement strategies across the full spectrum of research activities. |
| de Wit et al | Do not forget the professional – the value of the FIRST model for guiding the structural involvement of patients in rheumatology research. | 2015 | Netherlands | Authors apply the FIRST model as a framework to engage arthritis patients in research, and then, based on their experience, suggest opportunities for broadening the model to include additional recommendations. |
| De Wit et al | Patient participation as an integral part of patient-reported outcomes development ensures the representation of the patient voice: a case study from the field of rheumatology. | 2015 | Europe | Authors reflect on the role, contributions and representation of patient participants in the PsAID [Psoriatic Arthritis Impact of Disease] research process and present the lessons learned. |
| Deverka et al | Stakeholder participation in comparative effectiveness research: defining a framework for effective engagement. | 2012 | US | Aims to define stakeholder engagement and present a conceptual model for involving stakeholders in comparative effectiveness research, based on literature review, expert input and experience with the Center for Comparative Effectiveness Research in Cancer Genomics. |
| Dewar et al | Beyond tokenistic involvement of older people in research – a framework for future development and understanding. | 2005 | UK | Details a program concerning the development, implementation and evaluation of education that enables older people to feel more confident in partnership working. |
| Duffet | Patient engagement: What partnering with patient in research is all about | 2017 | Canada | Author provides an overview of patient engagement in research including levels of engagement, effective methods for engagement (best practices) and examples of patient engagement in research. Also focuses on the how patient engagement can apply to venous thromboembolism research. |
| Fagan et al | Implementing a pragmatic framework for authentic patient–researcher partnerships in clinical research. | 2016 | US | Authors identify several issues with available models for engaging research stakeholders, describe their own framework of collaborative engagement between investigators and research stakeholders, and identify determinants that enable this research approach to be effective at academic medical centers. |
| Fairbrother et al | Involving patients in clinical research: the Telescot Patient Panel. | 2013 | UK | Outlines the approach taken to patient involvement in the Telescot feasibility trial, considers the impact of the approach, and presents key learning points to assist clinical researchers who wish to engage patients in research development. |
| Forsythe et al | Patient and Stakeholder Engagement in the PCORI Pilot Projects: Description and Lessons Learned. | 2016 | US | Characterizes patient and stakeholder engagement in 50 Pilot Projects funded by the Patient-Centered Outcomes Research Institute (PCORI), and identifies early contributions and lessons learned. |
| Forsythe et al | A Systematic Review of Approaches for Engaging Patients for Research on Rare Diseases. | 2014 | US | Authors systematically study strategies used by rare disease organizations to facilitate patient engagement in research. |
| Frank et al | Conceptual and practical foundations of patient engagement in research at the patient-centered outcomes research institute. | 2015 | US | Presents PCORI’s conceptual model for patient-centered outcomes research [PCOR], summarizes funding requirements, reviews efforts to understand engagement in research among awardees, patients and clinicians, and outlines the development of the Engagement Rubric that guides engagement at PCORI. |
| Guise et al | A practice-based tool for engaging stakeholders in future research: a synthesis of current practices. | 2013 | US | A review of engagement methods utilized by academic institutions, practice-based research networks, NPOs, other organizations devoted to healthcare issues in US, Canada and Europe, leading to recommendations and best practices for stakeholder engagement. |
| Hewlett et al | Patients and professionals as research partners: Challenges, practicalities, and benefits. | 2006 | UK, Norway | Reports the combined experiences of researchers and patients who are collaborating in rheumatology research, and the working model that has evolved, providing one example of a practical model for collaboration based on experience. |
| Hoffman et al | How Best To Engage Patients, Doctors, And Other Stakeholders In Designing Comparative Effectiveness Studies. | 2010 | US | Proposes suggestions for meaningful stakeholder engagement, developed via a qualitative assessment of ongoing projects at the Center for Medical Technology Policy, with the intent to provide a framework to describe and organize this type of engagement. |
| INVOLVE (National Institute for Health Research) | Briefing notes for researchers: Public involvement in NHS, public health and social care research | 2012 | UK | A series of 10 briefing notes for researchers about public involvement in researcher. The information helps plan, resource and support public involvement in research. |
| INVOLVE (National Institute for Health Research) | Values, principles and standards for public involvement in research | 2013 | UK | Reports the findings of a review where public involvement values and summary principles are described for the first time by this group. |
| INVOLVE (National Institute for Health Research) | Public involvement in research: Values and principles | 2015 | UK | A revised version of the values and principles published in 2013. The revised versions reflect feedback from stakeholders and conceptual changes to the original set of values. |
| Israel et al | Challenges and Facilitating Factors in Sustaining Community-Based Participatory Research Partnerships: Lessons Learned from the Detroit, New York City and Seattle Urban Research Centers. | 2006 | US | Uses the experiences of three urban research centers to examine methods for sustainability of community-based participatory research [CBPR], employing a “lessons-learned” model. |
| James et al | Community ACTION Boards: An Innovative Model for Effective Community-Academic Research Partnerships. | 2011 | US | Shares lessons learned and practical strategies/tools for achieving success in developing partnership models, based on authors’ experience developing the East and Central Harlem Health Outcomes community advisory board [CAB]. |
| Jenner et al | Practical considerations in improving research through public involvement. | 2015 | UK | Presents a practical approach to bringing academic and patient communities together to support a portfolio of clinical research studies and provides insights from the viewpoint of both patient and researcher. |
| Jinks et al | Patient and public involvement in primary care research - an example of ensuring its sustainability. | 2016 | UK | Uses the case of the Primary Care Research Centre to provide a historical account of the evolution of patient and public involvement [PPI] in the Centre and identifies a number of key conceptual issues regarding infrastructure, resource allocation, working methods, roles and relationships. |
| Johnson et al | The patient voice in research—evolution of a role. | 2016 | US | Shares, based on author experiences, how the patient voice can affect the execution of a research study and provides a model for meaningfully engaging patients in research. |
| Kirwan et al | Emerging guidelines for Patient Engagement in Research | 2017 | UK | Authors describe and discuss different experiences integrating patients as research partners in outcomes research and draw from real-world examples. They suggest basic guidelines for researchers who are seeking to partner with patents. |
| Langston et al | An integrated approach to consumer representation and involvement in a multicentre randomized controlled trial. | 2005 | UK | Authors develop a template for other researchers/organizations to inform who becomes involved and how to best promote collaboration with consumers, based off their successful experience (up to level of disseminating results). |
| Lavallee et al | Stakeholder engagement in patient-centered outcomes research: high-touch or high-tech? | 2014 | US | Compares and contrasts high-tech and high-touch approaches to engaging stakeholders and suggest hybrid processes. |
| Lavallee et al | Initiative to support patient involvement in research | 2016 | USA | Authors report findings from a qualitative study involving researchers and patient/caregiver partners. 37 interviews were conducted. The report highlights the structure of these partnerships, important skills for researchers and patient partners as well as tools, resources and training suggestions. |
| Lavallee et al | Stakeholder engagement in comparative effectiveness research: how will we measure success | 2012 | USA | Authors propose a evaluation plan of patient stakeholder engagement in research. The report potential outcomes to assess and present a sex meta-criteria for evaluation. |
| Lindenmeyer et al | Assessment of the benefits of user involvement in health research from the Warwick Diabetes Care Research User Group: a qualitative case study. | 2007 | UK | Using the Warwick Diabetes Care research project and associated user group, assesses the benefits of involving health care users in diabetes research, and, through interviews of researchers and research users, comments on effective strategies of user-researcher collaboration and addresses what makes user involvement successful, effective and meaningful. |
| Madrid et al | Sharing Experiences and Expertise: The Health Care Systems Research Network Workshop on Patient Engagement in Research. | 2016 | US | Describes The Health Care Systems Research Network’s (HCSRN) Patient Engagement in Research Scientific Interest Group (PER SIG) work-shop, developed by researchers and patient partners with topics including: engagement of patient partners in developing research studies, nurturing partnerships, and assessing the impact of patient engagement in research |
| Marlett et al | Building new roles and relationships in research: a model of patient engagement research. | 2015 | Canada | Details the emergent role of patient engagement researchers as part of internship to prepare patients to take up new research roles within Alberta’s SCNs. |
| Marsden et al | Patient and clinician collaboration in the design of a national randomized breast cancer trial. | 2004 | UK | Shows that involving breast cancer patients in the design of a RCT studying hormone replacement therapy increases study accrual. As part of the study, authors set up an advisory board of patients who had early close interaction with the researchers who ultimately ran the study. |
| McAllister et al | Parents, Practitioners, and Researchers: Community-Based Participatory Research With Early Head Start. | 2003 | US | Reviews the underlying premise of community-based participatory research [CBPR] and emphasizes its effects, including increasing the capacity of researchers to identify, understand, and effectively address key public health issues. |
| McNeil et al | Engaging older adults in healthcare research and planning: a realist synthesis. | 2016 | Canada | Conducts a realist synthesis of the available knowledge on engagement in healthcare research and planning, informed by a framework following five phases including consultations with older adults. |
| Newman et al | Community Advisory Boards in Community-Based Participatory Research: A summary of Best Practices. | 2011 | US | Describes the best processes for forming, operating, and maintaining community advisory boards for community-based participatory research [CBPR]. |
| Nierse et al | Collaboration and co-ownership in research: dynamics and dialogues between patient research partners and professional researchers in a research team. | 2011 | The Netherlands | Presents a case study of collaboration and co-ownership between patient research partners and professional researchers. |
| Pandi-Perumal et al | Project stakeholder management in the clinical research environment: how to do it right. | 2015 | US, India | A conceptual framework for stakeholder management in the clinical research environment with practical suggestions for fostering meaningful stakeholder engagement, using the fifth edition of PMBOK® of the Project Management Institute as a basis for the highlighted guidelines. |
| Pandya-Wood et al | A framework for public involvement at the design stage of NHS health and social care research: time to develop ethically conscious standard. | 2017 | UK | An opinion piece informed by the authors’ first-hand experiences of providing advice on involving the public as part of the National Institute of Health Research (NIHR) Research Design Service (RDS) roles. |
| Perlmutter et al | Involving Advocates in Cancer Research. | 2015 | US | Takes examples of how patients/advocates are involved in different stages of research, then formulates a hypothesis about how to maximize effective engagement. |
| Rhodes et al | A service users’ research advisory group from the perspectives of both service users and researchers. | 2002 | UK | Service users' and researchers' perspectives on a service users’ advisory group set up to support and advise a project evaluating diabetes services. |
| Salsberg et al | Successful Strategies to Engage Research Partners for Translating Evidence into Action in Community Health: A Critical Review. | 2015 | Canada | A critical review describing key strategies supporting development of participatory research (PR) teams to engage partners for creation and translation of action-oriented knowledge. |
| Shalowitz et al | Community-Based Participatory Research: A Review of the Literature With Strategies for Community Engagement. | 2009 | US | Presents a guiding framework based on a model developed by the CDC in collaboration with Association of Schools of Public Health, highlighting best practices with examples for successful partnerships based on authors’ experiences. |
| Sheridan et al | The PCORI Engagement Rubric: Promising Practices for Partnering in Research. | 2017 | US | Description of how PCORI developed a framework for operationalizing stakeholder engagement in research, known as the PCORI Engagement Rubric. |
| Shippee et al | Patient and service user engagement in research: a systematic review and synthesized framework. | 2013 | US | Authors set out to delineate 1) integral patient and service user engagement [PSUE] components and 2) various stages at which PSUE can successfully occur. |
| Suarez-Balcazar et al | An Interactive and Contextual Model of Community-University Collaborations for Research and Action. | 2005 | US | Proposes an interactive and contextual model for developing and sustaining community-university partnerships, detailing three phases: gaining entry into the community, developing and sustaining a mutual collaboration, and recognizing the benefits and outcomes of partnership work. Also addresses potential challenges that might threaten these partnerships. |
| Supple et al | From tokenism to meaningful engagement: best practices in patient involvement in an EU project. | 2015 | UK | Comments on patient involvement in one of the biggest EU projects to date—U-BIOPRED, describing how people and carers of people with asthma have been able to drive input in disease-related research, and develops five key principles for the success of patient involvement. |
